# Clinical Experience of Intra-tumoral Central-Dose Escalated Volumetric Modulated Arc Therapy for Lymph Node Metastases in Patients With Advanced Cancer

**DOI:** 10.7759/cureus.34995

**Published:** 2023-02-14

**Authors:** Masateru Fujiwara, Fuminori Kitada

**Affiliations:** 1 Radiation Oncology, Suita Tokushukai Hospital, Suita, JPN; 2 Gynecologic Oncology, Suita Tokushukai Hospital, Suita, JPN

**Keywords:** lymph node metastasis, karnofsky performance scale (kps), dose-escalation, central-dose, advanced cancer, lymph node metastases, lymphnode metastasis (lnm), imrt, vmat, volumetric-modulated arc therapy

## Abstract

Background

Lymph node metastases (LN mets) are radioresistant, and high-dose irradiation is preferred for their control. The volumetric-modulated arc therapy technique makes it possible to perform intra-tumoral dose escalation without increasing the total prescribed dose of fractionated irradiation. We report its clinical experiences with intra-tumoral central-dose escalated volumetric-modulated arc therapy (ICE-VMAT) for LN mets.

Materials and methods

This study retrospectively evaluated 31 patients with 50 LN mets from stage III and IV advanced cancers who received ICE-VMAT. The total described dose was 50 Gy, and the median intra-tumoral central dose was 66 Gy (range, 54-79 Gy).

Results

The median follow-up period was 21 months. The two-year local control and overall survival (OS) rates were 95% and 56%, whereas univariate analysis revealed that the KPS ≥ 80 group had a significantly better OS compared to the KPS < 80 group.

Conclusion

ICE-VMAT was effective for LN mets. Patients with good KPS may benefit from therapeutic intervention with ICE-VMAT, even if they have multiple distant LN mets.

## Introduction

With the recent advances in chemotherapy, more patients with locally advanced diseases or distant metastases have achieved long-term survival [1]. Given the increasing number of patients with cancer achieving long-term survival, maintaining good performance status by improving treatment efficacy and reducing long-term side effects in patients previously considered to be terminally ill has become a challenge.

Recently, in radiation therapy (RT), the technology of volumetric-modulated arc therapy (VMAT) has made it possible to improve local control by increasing the dose concentration and reducing the side effects [2]. This development of radiation therapy technology has led to aggressive treatment of metastatic lesions in patients with advanced cancer. Several studies have recognized the efficacy of a local aggressive intervention in patients with oligometastases, even in patients with distant metastases. [3-5]. Even in the cases of lymph node metastases (LN mets), the efficacy of aggressive therapeutic intervention against oligometastases has been reported [6-10]. Recent genomic analyses of the treatment of LN mets have shown that susceptibility to LN mets and control by RT are regulated by genes rather than the primary site [11, 12]. Based on this concept, several prospective studies have not classified lymph nodes by primary site. [3-5]. LN mets have been noted to be radioresistant due to hypoxic environments in the center of the tumor, and a dose equivalent to or higher than the curative dose may be required to control the tumor [13-15]. However, given that LN mets in the trunk are often adjacent to organs at risk (OARs), administering medical treatment while reducing side effects has remained problematic. LN mets are filled with cancer cells and do not contain OARs. Therefore, irradiation using a dose profile with an escalating radiation dose in the intra-tumoral central area and a sharply dropping dose in the lymph node edges, commonly used in stereotactic radiation therapy (SBRT), could reduce damage to surrounding organs and improve tumor control. This method is applicable not only to SBRT for oligometastatic LN mets but also to conventional fractionated RT when prophylactic radiation is irradiated simultaneously for multiple LN mets or when SBRT is not possible due to large tumors. We considered that this method made it possible to improve the therapeutic effect and reduce side effects by performing the escalation of intra-tumoral irradiation dose without increasing the prescribed total dose in conventional fractionated RT. We call this planning concept "intra-tumoral central dose escalation" and this irradiation method "intra-tumoral central dose escalated volumetric-modulated arc therapy" (ICE-VMAT).

## Materials and methods

Patients

This retrospective study was conducted in accordance with the tenets of the Declaration of Helsinki and approved by the institutional review board at the Tokushukai Group Ethics Committee (TGE02094-071). A total of 31 patients with 50 LN mets of stage III and IV advanced cancer who were treated with intra-tumoral central-dose escalated volumetric-modulated arc therapy (ICE-VMAT) using a TrueBeam STx (Varian Medical Systems, Palo Alto, CA, USA) between 2017 and 2021 are included herein. ICE-VMAT was used in cases in which radical stereotactic radiotherapy (SBRT) could not be applied due to the large tumor size, concurrent irradiation of regional lymph nodes, or multiple metastases outside of the irradiation field. In this study, LN mets with a minor diameter greater than 10 mm within the irradiated field were treated with ICE-VMAT. All patients included in this study provided informed consent prior to treatment.

The patient characteristics are summarized in Table 1. The patients had a median age of 66 years (range, 33-82 years). Seven (23%), five (16%), seven (23%), and 12 (38%) of the patients had a Karnofsky Performance Status (KPS) score of 100, 90, 80, and ≤70, respectively. The primary sites included the nasopharynx (one patient), esophagus (one patient), lungs (five patients), breast (one patient), pancreas (one patient), colorectum (five patients), bladder (two patients), ureter (two patients), prostate (four patients), and female reproductive organs (nine patients). In this study, all primary sites had been controlled by surgery (61%) or radiotherapy (39%) within the follow-up period. And three of all patients (10%) had their primary tumor treated concurrently with LN mets. Irradiated 50 lymph nodes were localized in the mediastinum (15 tumors), axilla (one tumor), hepatic hilum (one tumor), para-aortic (17 tumors), and pelvic areas (16 tumors), of which 25 (50%) were distant metastases. A total of 18 (58%) had multiple metastases. Excluding the irradiated lymph node area, one of these patients had multiple brain metastases; five had multiple lung metastases; five had multiple liver metastases; two had multiple lung and liver metastases; and five had multiple LN metastases outside the irradiation field. And a total of 17 (55%) patients had symptoms that were thought to be due to LN mets. Chemotherapy was used in combination to treat 33 LN mets (66%).

**Table 1:** Patients’ characteristics ^a^ No. of pts.: Number of patients; ^b^ Value is presented as number (%) or median (range); ^c^ KPS: Karnofsky Performance Status; ^d^ Oligo was defined as up to five metastatic lesions with a controlled primary tumor

Variable		No. of pts.^a^ (%)^b^
Age (years)	Median (range)	66 (33-82)
Gender	Male	13 (42)
	Female	18 (58)
KPS^c^	100	7 (23)
	90	5 (16)
	80	7 (23)
	≤70	12 (38)
The original primary site	Nasopharynx	1 (3)
	Esophagus	1 (3)
	Lung	5 (16)
	Breast	1 (3)
	Pancreatic	1 (3)
	Colorectal	5 (16)
	Bladder	2 (7)
	Ureter	2 (7)
	Prostate	4 (13)
	Uterine cervix	6 (19)
	Uterine corpus	2 (7)
	Overy	1 (3)
Distant metastases	Yes	18 (58)
	No	13 (42)
Metastatic status	Oligo^d^	13 (42)
	Multiple	18 (58)
Associated symptoms	Pain	13 (42)
	Cough	4 (13)
	Asymptomatic	14 (45)

Radiation Therapy

**Figure 1:**
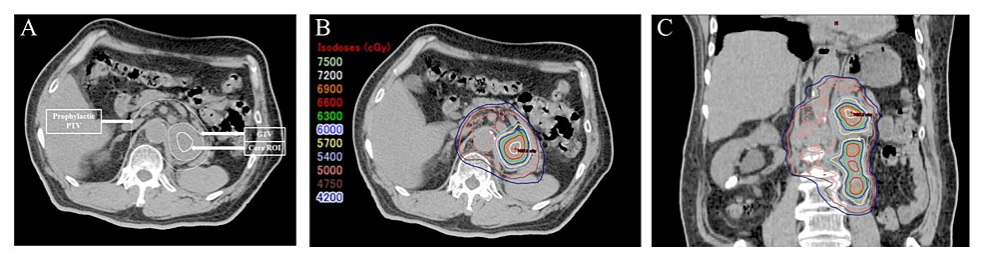
Example of ICE-VMAT Example of a region of interest (ROI) configuration (A) for a patient with para-aortic LN mets and the axial image (B) and sagittal image (C) of the intra-tumoral central dose escalated volumetric-modulated arc therapy plan with simultaneous boost irradiation by intra-tumoral central-dose escalating given to four LN mets showing isodose distribution.

All patients underwent VMAT using TrueBeam STx. After immobilization using Vac-Lok positioning cushions (CIVCO Medical Solutions, CA, USA) as a vacuum pillow, a free-breath CT for treatment planning was performed. The gross tumor volume (GTV) was defined as lymph node metastatic tumors. The clinical target volume (CTV) was created by adding selective prophylactic lymph stations to GTV. The planning target volume (PTV) was determined by adding 4-5 mm set-up margins to the CTV. ICE-VMAT plans were created using the Eclipse treatment planning system (version 11.0, Varian Medical Systems) with the Acuros XB algorithm for heterogeneity correction. The core region of interest (Core-ROI) was created 3-8 mm inside the GTV margin to optimize the VMAT plan in order to escalate the intra-tumoral central dose (Figure 1A). The treatment plan was optimized using the simultaneous integrated boost (SIB) method so that the dose gradient inside the GTV was increased at 3-5% per mm toward the center of the Core-ROI. And within the Core-ROI, the intra-tumoral central dose of the GTV was escalated up to approximately 125%-140% of the prescribed dose for the PTV. In addition, the plans ensured that GTV D99 (the radiation dose received by 99% of the GTV) was irradiated for more than 95% of the total prescribed dose. Treatment plans were generated and delivered based on 6- or 10-megavolt (MV) photon beams from a TrueBeam STx linear accelerator equipped with 2.5-mm multi-leaf collimators. The figure shows the dose distribution map of ICE-VMAT (Figure 1B and Figure 1C).

The characteristics of the treatment plans are listed in Table 2. ICE-VMAT with the dose of 50 Gy prescribed to PTV D50 (the radiation dose received by 50% of the PTV) was used to treat LN mets. The median dose per fraction was 2.5 Gy (range, 2.0-2.5 Gy), whereas the median intra-tumoral central dose defined as the maximum dose within Core-ROI was 66 Gy (range, 54-79 Gy), which was 70 Gy (range, 57-87 Gy) converted to an equivalent dose in a 2 Gy fraction with the α/β ratio of 10 (EQD2_10_). EQD2_10_ was calculated using the linear quadratic (LQ) equation for each schedule. The equation used to calculate the EQD2_10_ was as follows:

EQD2_10_ = Nd (d+α/β) / (2+α/β) = Nd (d+10) / (2+10)

N is the fraction number of RT, and d is the fractional dose of RT. For the LQ calculation, a value of α/β = 10 was assumed for tumors.

Patient alignment was performed through image-guided radiation therapy (IGRT) with cone-beam computed tomography (CBCT) imaging each time. ICE-VMAT plans were replanned during the RT at 5 (10%) of all LN mets due to tumor volume reduction.

**Table 2:** Treatment targets’ characteristics ^a^ Value is presented as a number (%) or median (range); ^b^ EQD2_10_: equivalent dose in 2 Gy fraction with the α/β ratio of 10

Variable		No. of targets (%)^a^
Irradiated lymph node location	Mediastinum	15 (30)
	Axilla	1 (2)
	Hepatic hilum	1 (2)
	Para-aortic	17 (34)
	Pelvic	16 (32)
Tumor volume (cm^3^)	Median (range)	6 (1-304)
Dose per fraction (Gy)	Median (range)	2.5 (2.0-2.5)
Central dose (Gy)	Median (range)	66 (54-79)
Central dose converted to EQD2_10_^ b^(Gy)	Median (range)	70 (57-87)
Concurrent chemotherapy	Yes	33 (66)
	No	17 (34)
Initial effect	Complete response	48 (96)
	Partial response	2 (4)

Evaluation of Clinical Outcomes

In principle, patients undergo contrast-enhanced CT after four weeks and then every three months thereafter. The last visit or date of contact was used to censor surviving patients at the time of analysis, after which overall survival (OS) was evaluated. Tumor response was evaluated using the Response Evaluation Criteria in Solid Tumors (version 1.1) [16]. Complete response, partial response, and stable disease were categorized as local control (LC), whereas progressive disease was categorized as a local failure. Adverse events after RT were evaluated using the National Cancer Institute’s Common Terminology Criteria for Adverse Events, version 5.0.

Statistical analysis

OS and LC rates were actuarially calculated using the Kaplan-Meier method. OS was defined as the duration from the first day of irradiation to death or the last follow-up. Local control was defined as the duration from irradiation to the date of local relapse. Univariate analyses were performed using the log-rank test to determine prognostic factors for OS or LC. Statistical analyses were performed using JMP Pro Software version 14.0 (SAS Institute Inc., Cary, NC, USA), with p < 0.05 indicating statistical significance.

## Results

**Figure 2:**
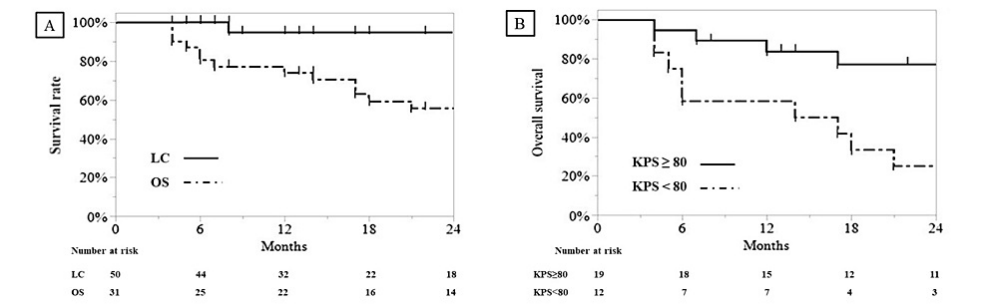
Survival curves Kaplan–Meier curves for the local control (LC) and overall survival (OS) rates of all patients (A) Overall survival rates according to Karnofsky Performance Status (KPS) Patients with KPS ≥ 80 (thick line) showed better OS rates compared to those with KPS < 80 (dotted line) (p < 0.001) (B).

The median follow-up period for surviving patients was 21 months (ranging from four to 54 months) after ICE-VMAT. Among the 50 LN mets, 48 and two showed a complete and partial response, respectively. Symptoms considered to have been due to LN mets showed improvement in all cases. LC after one and two years was 95%, whereas OS after one and two years was 74% and 56%, respectively (Figure 2A).

Regarding the toxicity associated with RT, a grade one vertebral compression fracture occurred in one case (3%), but no toxicity greater than grade two was noted during the observation period.

As shown in Table 3, univariate analysis revealed that the Karnofsky Performance Status (KPS) score was a significant prognostic factor for OS. Accordingly, patients with a KPS ≥ 80 showed better OS (1- and 2-year OS of 84% and 77%, respectively) compared to patients with a KPS < 80 (1- and 2-year OS of 50% and 17%, respectively) (hazard ratio = 0.16, p <0.001) (Figure 2, B). Overall, good KPS scores correlate with better OS.

**Table 3:** A univariate analysis of prognostic factors after the irradiation about overall survival ^a^ No. of pts: Number of patients; ^b^ Oligo-metastases were defined as up to 5 metastatic lesions with a controlled primary tumor

Variable	Strata	No. of pts. (%)^a^	p-value
Age (years)	>70	12 (39)	0.7134
	≤70	19 (61)	
Gender	Male	13 (42)	0.6924
	Female	18 (58)	
KPS	≥80	19 (62)	0.0003
	<80	12 (38)	
Original primary site	Pelvis	21 (68)	0.1615
	Without pelvis	10 (32)	
Chemotherapy use	Yes	21(68)	0.6663
	No	10(32)	
Distant metastases	Yes	18 (58)	0.0901
	No	13 (42)	
Oligo-metastases^b^	Yes	13 (42)	0.1205
	No	18 (58)	

## Discussion

Aggressive RT for LN mets has been mainly reported to be effective as a radical treatment for oligometastasis using SBRT. Generally, SBRT for LN mets has two-year LC rates of 47%-82% [6-10]. In this study, although 16 patients (52%) continued chemotherapy after ICE-VMAT, they showed the same excellent local control as SBRT. On the other hand, prospective studies of SBRT for oligometastases from various primary sites by Salama et al., Milano et al., and Palma et al. have shown two-year survival rates of 57%, 50%, and 69%, respectively [3-5]. And, retrospective studies for LN oligometastases by Wang et al., Yeng et al., Loi et al., Ito et al., and Shahi et al. have shown two-year survival rates of 50%, 74%, 65%, 63%, and 64% [6-10]. Although more than half of our cases presented with multiple metastases, our results were comparable to those of oligometastases reported previously. This is largely due to the recent advances in chemotherapy as well as improvements in the systemic anticancer effects of chemotherapy and the reduced side effects of adjuvant therapies. The rate of adverse events of SBRT for LN mets was reported to be 0%-3% in grades three or higher, but none were observed in grades two or higher in our study, which was safer in comparison [6-10].

This study also showed that KPS was a significant prognostic factor for OS, whereas the presence of multiple and distant metastases was not. KPS is an index introduced by Karnofsky et al. to evaluate the general condition at the start of chemotherapy [17]. Buccheri et al. have demonstrated the importance of KPS as a prognostic factor in patients receiving chemotherapy for lung cancer [18]. Therefore, we believe that patients with advanced cancer disease who have good KPS scores and can tolerate aggressive chemotherapy should receive aggressive local control with RT in combination with chemotherapy for individual lesions that are difficult to control with chemotherapy alone.

However, the occurrence of serious adverse effects of RT can certainly impair the patient’s general condition and interfere with the continuation of chemotherapy. Therefore, this study used the ICE-VMAT irradiation method to plan for a substantial increase in intra-tumoral dose while maintaining a low dose toward the surrounding OARs. A report by Oku et al. showed that when the de-escalation of the prescription isodose line reaches 60% of the central dose during stereotactic body radiotherapy, the central dose can be increased while maintaining a reduced dose to the OARs around the treatment target. Moreover, given that a rapid dose gradient can be maintained, the treatment target dose can be significantly increased without increasing the total dose [19, 20]. Takeda et al. reported its clinical usefulness in lung cancer and liver cancer [21, 22]. Chan et al. also reported that a similar treatment plan could be replicated with VMAT [23]. The aforementioned reports have certainly promoted the current increase in the utilization of the central dose-escalation method in SBRT. Moreover, the ICE-VMAT utilized in the current study is based on this concept. When the treatment targets are uniformly composed of tumors, ICE-VMAT should be performed after ensuring the accuracy of positioning and fixation through IGRT in every conventional fractionated irradiation for multiple or large LN mets, for which extreme hypofractionated SBRT is not applicable. LN mets, in particular, are good treatment targets for ICE-VMAT, with this study showing few adverse events and excellent local control.

Limitations

This study had several limitations. Firstly, it was a retrospective analysis using observational data. Secondly, there was the inclusion of a heterogeneous group of patients with different primary tumors, different LN mets treatment sites, disease extent, and systemic therapies used. Thirdly, as the sample size was small, it was premature to generalize our findings, especially in terms of prognostic factors. Larger retrospective cohorts and prospective clinical studies are likely required to identify a subset of patients with lymph node metastases disease who benefit the most from ICE-VMAT.

## Conclusions

In conclusion, our results showed the potential benefits and safety of the ICE-VMAT for LN mets. Moreover, our analysis identified KPS as a significant prognostic factor for OS in patients with LN mets of advanced cancers. Therefore, patients with a good KPS may benefit from therapeutic intervention with ICE-VMAT, even if they have multiple distant LN mets.
